# The effect of passion on the engagement of university students specializing in DanceSport: the moderating role of gender

**DOI:** 10.3389/fpsyg.2025.1615585

**Published:** 2025-07-09

**Authors:** Ziyou Huang, Jiamin Zhu, Yuxin Yuan, Xiaofen Li

**Affiliations:** ^1^School of Physical Education, Anqing Normal University, Anqing, China; ^2^Graduate School of Shandong Sport University, Jinan, China; ^3^School of Arts, Beijing Sport University, Beijing, China

**Keywords:** passion, engagement, DanceSport, gender, moderating effect

## Abstract

**Objectives:**

This study aims to investigate the effect of passion on DanceSport engagement (DSE) of college students majoring in DanceSport, and to examine the moderating effect of gender.

**Methods:**

We investigated 714 college students majoring in DanceSport with Passion Scale and Athlete Engagement Questionnaire from major sports institutes in China and Beijing Dance Academy.

**Results:**

Passion was positively correlated with DSE (*r* = 0.651, *p* < 0.01). Among college students specializing in DanceSport, training time shows a significant positive correlation with education level, gender, passion, and DSE (*p* < 0.05). However, education level and gender had no correlation between passion and DSE (*p* > 0.05). Hierarchical regression analysis indicates that passion had a positive predictive effect on DSE (β = 0.677, *p* < 0.001). Gender had a positive moderating effect in the relationship between passion and DSE (β = −0.258, *p* < 0.001). Simple slope analysis revealed that the predictive effect was stronger for males (*simple slope* = 0.791) than for females (*simple slope* = 0.533) (*p* < 0.001).

**Conclusion:**

The discovery of the gender moderation effect broadened the dual model of passion, suggested that passion mechanisms differ based on the gendered nature of sports, offering a theoretical basis for nurturing DanceSport talent. For boys, enhancing DSE can involve boosting goal motivation for technical breakthroughs, increasing emotional expression training, and organizing simulated competitions. For girls, creating a cross-school dancer resource pool, implementing a “dual selection” mechanism, and boosting artistic expression and autonomy support can help overcome structural barriers and promote DSE.

## 1 Introduction

DanceSport, which integrates competitiveness, artistry, complexity, and procedure, requires male and female partners to achieve a harmonious combination of physical techniques and artistic expression within defined musical and rhythmic boundaries (Li, 2016). For university students majoring in DanceSport, their primary tasks involve theoretical learning, technical training, and competing in DanceSport. Ericsson et al. found that developing knowledge structures and skills in a specific field requires extensive deliberate practice (Ericsson et al., 1993; Ericsson, 1996). DanceSport is no exception; mastering solo techniques, partner coordination, and artistic expression necessitates thousands, if not tens of thousands, of hours of training to achieve competitive performance levels. This lengthy and repetitive technical training challenges the engagement levels of university students specializing in DanceSport (Ziyou, 2023).

Lonsdale defines athlete engagement as a positive, enduring psychological state characterized by confidence, dedication, vitality, and enthusiasm (Lonsdale et al., 2007). This engagement is crucial for sustaining training adherence and enhancing competitive performance (Zhang, 2012), reflecting a strong psychological identification with and active commitment to their sport (Lodah and Kejner, 1965). Higher levels of DanceSport engagement (DSE) among university students correlate with a greater willingness to continue participating in DanceSport practice. Research indicates that an individual’s level of DSE significantly influences their competitive performance (Liu et al., 2023). Furthermore, China’s 14th Five-Year Plan for Building a Strong Sports Nation (General Administration of Sport of China, 2021) underscores the urgency of advancing practical engagement and theoretical frameworks across sports to elevate educational and competitive standards by its culmination in 2025. Consequently, investigating the determinants and mechanisms of DSE among university DanceSport students holds strategic importance for talent development and the sport’s trajectory in China.

Self-Determination Theory posits that internalizing activities as part of one’s self-identity fosters autonomous motivation, which in turn sustains engagement (Ryan and Deci, 2000; Ryan and Deci, 2020). Passion, defined as a behavioral tendency arising from internalized enjoyment of an activity, drives individuals to invest time and effort in pursuits they value (Vallerand et al., 2003). In sports contexts, passion sustains individuals’ energy reserves during athletic participation (Lafrenière et al., 2012), and stimulates deliberate practice, thereby enhancing engagement and optimal performance (Vallerand et al., 2008). Research indicated that passion was a key antecedent of sustained engagement (Parastatidou et al., 2012; Carbonneau et al., 2010). Our Recent empirical studies have demonstrated that passion positively predicts DSE levels among university DanceSport students, facilitating technical mastery and artistic interpretation (Ziyou et al., 2024).

As a dyadic sport requiring male–female partnerships, DanceSport derives its esthetic essence from the synergistic interplay of partners’ technical and artistic execution. However, China’s DanceSport community contends with pronounced gender imbalances, evidenced by disproportionately low male participation. For instance, Beijing Sport University’s 2020 DanceSport cohort comprised only two male students, this disparity replicated nationwide. This gender imbalance frequently compels female dancers to abandon competitive aspirations due to partner shortages (Ziyou et al., 2024). Male dancers, who traditionally assume leadership roles in partnerships, face heightened performance expectations and demands for higher technical standards. China’s distinct sociocultural norms further amplify psychological pressures on DanceSport students of different genders, warranting rigorous exploration of gender’s moderating role in engagement dynamics.

Currently, DanceSport research predominantly examines DanceSport techniques (Chen et al., 2020), competitions (Li and Zhang, 2022; Wang et al., 2021), competitive performance (Liu X. et al., 2024), and injuries (Liu Z. et al., 2024). However, the psychological mechanisms through which passion and DSE serve as critical antecedents to sustained training adherence, skill mastery, and competitive achievement have received limited scholarly attention. Notably, the moderating role of gender in the passion-DSE relationship remains insufficiently investigated, particularly within the context of DanceSport’s structural gender disparities.

Therefore, this study adopts a gender lens to investigate how passion differentially influences DSE among male and female university DanceSport students, offering theoretical insights for talent development in DanceSport and other gender-imbalanced sports. Based on these, the study proposes the following hypotheses: (1) Passion positively predicts DSE. (2) Gender plays a moderating role between passion and DSE.

## 2 Methods

### 2.1 Participants

To ensure sample representativeness, a judgmental sampling strategy was adopted, prioritizing institutions with established DanceSport programs, students’ competitive proficiency, and systematic training duration. Participants were recruited from Beijing Dance Academy and eight national sports universities specializing in DanceSport education, including Beijing Sport University, Shenyang Sport University, Wuhan Institute of Physical Education, Jilin Sport University, Xi’an Physical Education University, Chengdu Sport University, Nanjing Sport University, and Guangzhou Sport University. Data collection was administered via Questionnaire Star, a validated online survey platform. To mitigate order effects, scale items were randomized across respondents. A total of 729 questionnaires were distributed. To minimize sampling bias, inclusion criteria required: (1) prior experience with a fixed dance partner. (2) ≥ 2 years of systematic DanceSport training. (3) participation in provincial-level or national competitions within the preceding 12 months. After excluding questionnaires with incomplete demographic data, paradoxical choices, or regular answer, 714 valid responses were retained (97.9% validity rate). Participant demographics are summarized in Table 1.

**Table 1:** Demographic information of the participants (*n* = 714).

Variable	Classification	Frequency	Percent (%)
Gender	Man	323	45.2
	Female	391	54.8
Education level	Undergraduates	622	87.1
	Postgraduates	92	12.9
Training time	2 ~ 5 years	242	33.9
	6 ~ 9 years	257	36.0
	≥10 years	215	30.1

### 2.2 Materials

#### 2.2.1 Passion scale

We used the “Passion Scale” to Measure participants’ level of passion. The scale includes two dimensions: harmonious passion and obsessive passion, comprising a total of 12 items. Based on the theme of this study, we incorporated the term “DanceSport” into the scenarios, such as in the harmonious passion measurement item: “The new things that I discover with DanceSport allow me to appreciate it even more,” and in the obsessive passion measurement item: “I have a tough time controlling my need to do DanceSport.” A 5-point Likert scale was employed, ranging from “strongly disagree” to “strongly agree,” scored from 1 to 5. A higher score indicates a greater passion for DanceSport among specialized university students. Chinese scholar has already translated and tested the Passion Scale in Chinese (Qingbao, 2018). In this study, the Cronbach’s α coefficient of the Passion Scale was 0.89, and the results of the confirmatory factor analysis were χ^2^/df = 1.45, RMSEA = 0.03, GFI = 0.98, AGFI = 0.97, NFI = 0.98, indicating good reliability and validity of the scale.

#### 2.2.2 Athlete engagement questionnaire

We used the “Athlete Engagement Questionnaire” (Lonsdale et al., 2007) to measure the level of DSE of the participants. This questionnaire includes four dimensions: confidence, enthusiasm, dedication, and vitality, comprising a total of 16 items. Based on the theme of this study, we incorporated the term “DanceSport” into the scenarios, such as in the dedication dimension with the statement “I hope to achieve my goals in DanceSport through hard work,” and in the vitality dimension with “I feel energetic during DanceSport training and competitions.” A Likert 5-point scale was employed, ranging from “never” to “always,” corresponding to scores of 1 to 5. A higher score indicates a greater level of DSE in DanceSport among specialized college students. Chinese scholars (Wang et al., 2014) have already translated and tested the Athlete Engagement Questionnaire. In this study, the Cronbach’s α coefficient of the questionnaire was 0.97, and the results of the confirmatory factor analysis were χ^2^/df = 2.15, RMSEA = 0.04, GFI = 0.98, AGFI = 0.97, NFI = 0.98, indicating good reliability and validity of the Athlete Engagement Questionnaire.

### 2.3 Analysis strategy

Descriptive statistical analysis and correlation analysis were performed using SPSS 29.0. To test the moderating role of gender, hierarchical regression analysis was performed. Passion scores were mean-centered to mitigate multicollinearity, and gender was coded as a dummy variable (0 = male, 1 = female), with males as the reference category. Confirmatory factor analysis (CFA) was conducted in AMOS 29.0 to assess common method bias. Trait-only and method-factor models were compared by examining changes in model fit indices.

### 2.4 Common method bias

Many psychologists have recommended the introduction of the method factor test for common method bias because it is more accurate than the Harman single factor test (Xiong et al., 2012; Tang and Wen, 2020). Therefore, this study employs the”Control for Unmeasured Single Method Latent Factor” method to test for common method bias, constructing Confirmatory Factor Model 1 and a model (Model 2) that includes the method factor, and comparing the main fit indices of the two models. If the changes in △CFI and △TLI are less than 0.1, and the change in △RMSEA is less than 0.05, then there is no significant common method bias (Podsakoff et al., 2003; Wen et al., 2018). Model 1 has a χ^2^/df = 1.59, RMSEA = 0.03, TLI = 0.99, CFI = 0.99. Model 2 has a χ^2^/df = 1.73, RMSEA = 0.03, AGFI = 0.97, TLI = 0.98, CFI = 0.99. The results show that △CFI = 0.00, △TLI = 0.01 < 0.1, and △RMSEA = 0.00 < 0.05, indicating that there is no significant common method bias in the measurements.

## 3 Results

### 3.1 Correlation of each variable

The means, standard deviations, and correlation analysis of the main variables in this study are shown in Table 2. It can be seen from the table that the training time has a significant positive correlation with education level, gender, passion, and DSE (*p* < 0.05). Passion is significantly positively correlated with DSE (*r* = 0.651, *p* < 0.01). Neither education level nor gender have a relationship between passion and DSE (*p* > 0.05).

**Table 2:** Correlation analysis results, mean and standard deviation (*n* = 714).

Variables	Education level	Training time	Gender	Passion	DSE
Education level	1.000				
Training time	0.322^**^	1.000			
Gender	0.047	0.094^*^	1.000		
Passion	−0.025	0.123^**^	0.05	1.000	
DSE	0.025	0.126^**^	0.58	0.651^**^	1.000
M				3.987	4.017
SD				0.581	0.607

*indicates *p* < 0.05. **indicates *p* < 0.01. DSE, DanceSport engagement.

### 3.2 Moderating effect

According to the relevant analysis results, there is no significant correlation between gender and passion or DSE, indicating that gender is a suitable choice for a moderating variable. Therefore, we further examine whether gender has a moderating effect between passion and DSE. Based on the moderating effect testing method (Wen et al., 2005), we first center the passion variable and set gender as a dummy variable, with males as the reference group (male = 0, female = 1). Subsequently, after controlling for variables such as education level and training duration, we use hierarchical regression analysis to test the moderating effect of gender on the passion and DSE of college students specializing in DanceSport.

The results show (see Table 3) that in Model 2, after controlling for education level and training time, the positive predictive effect of passion on DSE is significant (β = 0.677, *p* < 0.001), while gender does not have a significant impact on DSE (*p* > 0.05). In Model 3, the interaction term between passion and gender has a significant predictive effect on DSE (β = −0.258, *p* < 0.001, *△R^2^* = 0.015).

**Table 3:** Analysis results of the moderating effect of gender (*n* = 714).

Variables	Model 1		Model 2		Model 3	
	β	t	β	t	β	t
Constant	3.867	45.723	3.806	57.591	3.815	58.429
Education level	−0.031	−0.44	0.052	0.953	0.033	0.615
Training time	0.072***	3.358	0.018	1.070	0.018	1.083
Passion			0.677***	22.617	0.791***	20.044
Gender			0.061	1.747	0.089*	2.553
Passion*Gender					−0.258***	−4.369
*R* ^2^	0.016		0.430		0.445	
△*R*^2^	0.016***		0.414***		0.015***	
F	5.866**		133.596***		113.422***	

Dependent variable: DanceSport engagement.

*indicates *p* < 0.05. **indicates *p* < 0.01. ***indicates *p* < 0.001.

**Figure 1:**
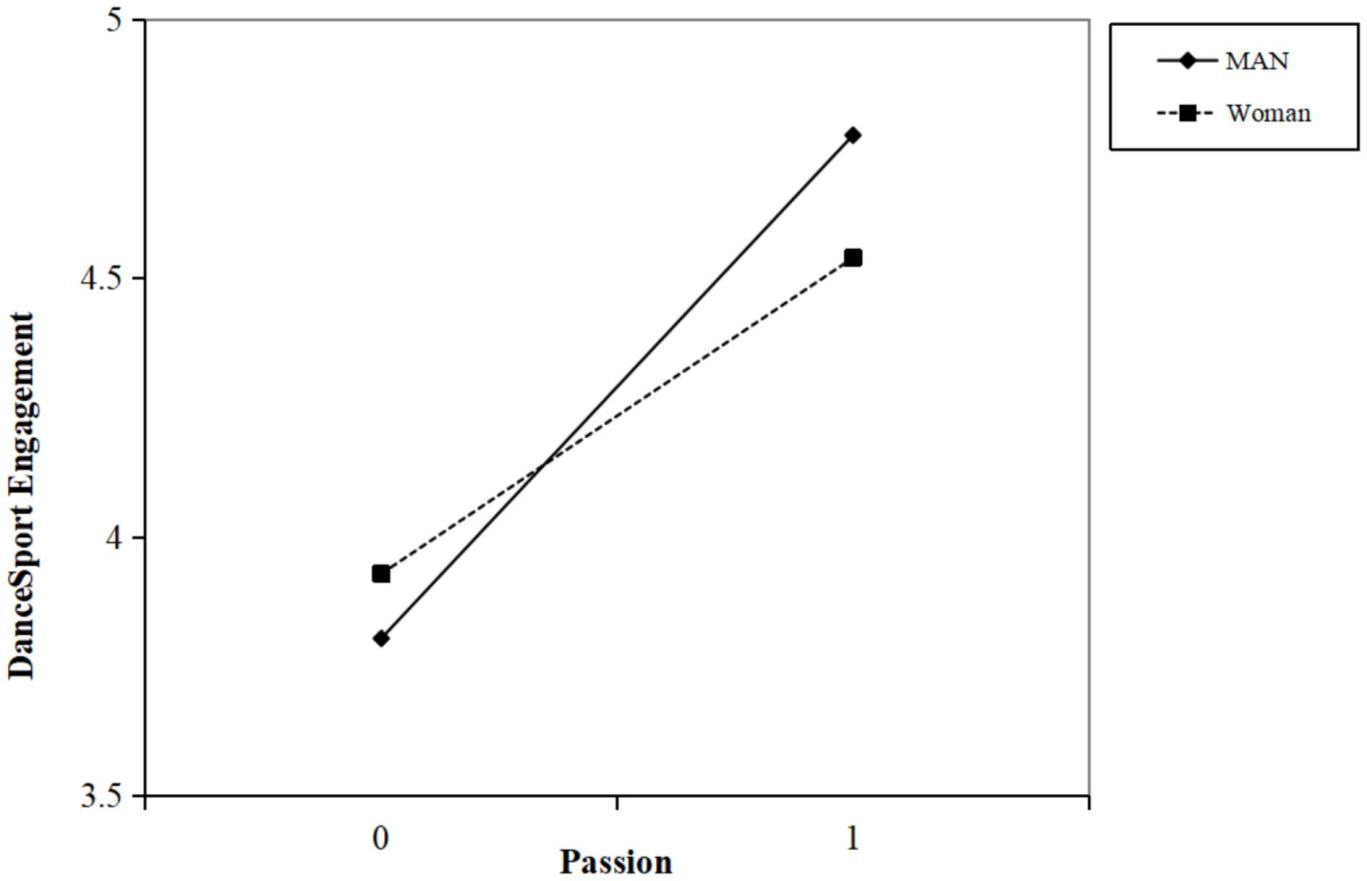
The moderating effect of gender on the passion and DSE.

To further examine the moderating effect of gender between passion and DSE, we plotted a simple slope plot (Figure 1). The slope of the line on the way reflects how much passion affects the DSE. Simple slope tests (Dearing and Hamilton, 2006) indicate that for males, as passion increases, there is a significant positive predictive effect on DanceSport DSE (*simple slope* = 0.791, *p* < 0.001). For females, as passion increases, there is also a significant positive predictive trend on DanceSport DSE (*simple slope* = 0.533, *p* < 0.001). However, compared to males, the predictive effect for females is relatively lower. Overall, the results indicate that the impact of passion on DSE is moderated by gender.

## 4 Discussion

This study explores the moderating role of gender in the relationship between passion and DSE among college students specializing in DanceSport. The main finding is that passion has a significant positive impact on DSE, and this relationship is moderated by gender: both male and female students experience an increase in DSE as their level of passion rises, but the predictive effect for female college students specializing in DanceSport is significantly lower than that for their male counterparts. Although additional variance explained is only 1.5% and the effect size is small (*△R^2^* = 0.015), the larger sample size (*n* = 714) and moderately large interaction term coefficients and strengths (β = −0.258) suggest that it may have theoretical significance.

On the one hand, the passion has a significant positive impact on the development of DSE among university students specializing in DanceSport, a finding that aligns with previous viewpoints (Oh, 2017). This supports the dual theory of passion in DanceSport. When students engage in DanceSport, they internalize it as part of their identity, which fuels passion and leads to higher engagement. Consequently, they tackle challenges in technique, artistic style, and competition with greater commitment (Ziyou et al., 2024). This is also consistent with the core tenets of self-determination theory (Ryan and Deci, 2000; Ryan and Deci, 2020), which emphasizes the role of intrinsic motivation and psychological needs (autonomy, competence, and relatedness) in engagement. By internalizing DanceSport as part of their identity, students fulfill autonomy (choosing dance styles), competence (improving skills), and relatedness (partner compatibility), enhancing DSE. It is evident that passion represents a positive high-level emotional state and behavioral attitude (Ho et al., 2011). In sports, individuals with high passion see activity value and are intrinsically motivated (Pertula and Cardon Cameron, 2011). This intrinsic drive can significantly enhance the state of engagement, encouraging individuals to invest more time and energy into the sports they love (Vallerand and Houlfort, 2019; Slemp et al., 2021).

On the other hand, gender moderates the effect of passion on DSE. Both male and female students show increased engagement as passion rises, but the effect is stronger for males. This mirrors the influence of gender on passion and perseverance (Sigmundsson et al., 2020). Additionally, as He et al. found, the relationship between passion and high potential is stronger for men than for women (He et al., 2024). This suggests that male students’ passion is more easily transformed into DSE. The fundamental reason for this phenomenon may be the unique project background and socio-cultural context of Chinese DanceSport. The gender imbalance, characterized by a higher number of male students, allows them to gain more attention from teachers. Additionally, the role of males as leaders in DanceSport imposes higher standards and requirements on their technical skills, training intensity, and adaptability in competitions, which may amplify their sense of responsibility and commitment when their passion is high, thereby reinforcing their DSE. In contrast, female students may experience a corresponding weakening of the transformation of passion into DSE due to limitations in gender-available resources, this is consistent with the idea proposed by Vallerand et al. that the maintenance, development, and transformation of passion are influenced by factors such as environment, culture, and interpersonal relationships (Vallerand, 2010; Mageau et al., 2009). As resource conservation theory suggests, when individuals perceive a lack of social resources (such as male dance partners or partner relationships), the link between motivation and behavior may be weakened, leading to a reduction in their engagement (Hobfoll, 1989).

Furthermore, from the perspective of self-determination theory (Ryan and Deci, 2000; Ryan and Deci, 2020), males in DanceSport more frequently assume a technically dominant role (such as lead dancer), which makes it easier for them to achieve a sense of competence; whereas female dancers, despite having comparable levels of passion, however, due to limited partner resources, the sense of belonging and autonomy may be weakened, leading to a diminished driving effect of passion on DSE.

Additionally, social role theory posits that societal roles generate behavioral expectations (Eagly, 2013). and gender differences arise from differential societal expectations (Bussey and Bandura, 1999). In DanceSport, men typically lead, while women follow. This division reinforces the view of men as technical experts and women as performers, intensifying men’s focus on skill development. Passion is more easily translated into persistence in training for men, while women may face a “decoupling” of passion and DSE due to dependency on male partners.

Gender schema theory further indicates that individuals internalize gender norms through the socialization process and are more likely to pay attention to, remember, and process information that is consistent with their own gender schema (Liben and Bigler, 2002; Bem, 1993). Male college students specializing in DanceSport may be more likely to activate a “goal-oriented” schema (such as personal technical breakthroughs), tending to associate passion with competition and achievement, thereby enhancing their dance experience. They are fully committed when the learning, training, and competition in DanceSport align with their personal goals. In contrast, females may be more inclined to activate a “relationship-oriented” schema (such as training with a partner), placing greater emphasis on emotional expression, which leads to differences in the predictive power of passion on DSE.

Finally, from a neurobiological perspective, this may be related to higher dopamine levels released in males during physical activity. Dopamine significantly influences goal motivation, attention, and behavior, promoting perseverance and engagement in exercise (Munro et al., 2006; Soutschek et al., 2017).

## 5 Conclusion

This study confirms that passion is a key driving factor in enhancing the DSE of college students specializing in DanceSport. It also finds that gender has a significant moderating effect between the two, with both male and female students’ engagement in sports increasing with heightened passion, and the predictive effect being stronger for males than for females. The discovery of the gender moderating effect expands the theoretical framework of the existing dual model of passion, indicating that the mechanism of passion may vary due to the gendered characteristics of the sport.

According to research findings, in promoting the transformation of passion into DSE of DanceSport students, teachers can adjust their motivational strategies based on gender differences.

For male students: (1) By designing training objectives that focus on individual techniques and partner coordination within DanceSport combinations, it is essential to strengthen their passion for technical breakthroughs and enhance their DSE. (2) Regular communication and interaction with partners should be encouraged to reinforce emotional expression training, compensating for the lack of relationship orientation. (3) Designing simulated DanceSport competitions can enhance the importance and sense of responsibility of male students as leaders in partner dances during these simulations, thereby increasing passion and the conversion rate of DSE. (4) The curriculum should frequently include segments for male students to showcase individually, which can elevate their immediate passion and facilitate the instant transformation of DSE.

For female students: (1) In response to the challenge of insufficient partners faced by female students, universities can establish a cross-institutional dancer resource pool or implement a”dual selection” mechanism to reduce the suppression of structural barriers on passion transformation. (2) By establishing incentive measures, enhance female college students’ passion for DanceSport to promote DSE. (3) Focus on artistic expression and support for autonomy to enhance the role of passion in promoting DSE. For the sport of dance: Promote the reform of the scoring system to balance the weight of technique and artistic expression, and reduce the limitations imposed by gender role rigidity on the development of dance participants.

## 6 Strengths and limitations

Strengths: (1) This study starts from the characteristics of typical male–female pairing in DanceSport and finds that gender moderates the impact of passion on DSE. This finding provides a theoretical contribution to the development of sports psychology research related to DanceSport and offers more targeted suggestions for improving the engagement levels of university students in sports based on gender. (2) The study validates the applicability of passion theory in DanceSport, expanding the theoretical framework of the dual model of passion. (3) The research explains the theoretical reasons for the differences in how different genders moderate the transformation of passion into DSE from various perspectives, including self-determination theory, social role theory, gender schema theory, and neurobiological mechanisms. It reveals that the mechanisms of passion vary due to the gendered characteristics of the sport, indicating that sports psychology needs to pay attention to the specificity of different sports.

Limitations: (1) The judgment sampling used in the study is determined according to the team’s decades of sports dance learning experience and college teaching experience. The selection of samples is generally in line with the distribution of college students majoring in sports dance in China, however, there may be a slight bias. Future studies can try to use a larger sample size or a more suitable sampling method. (2) All data measurements are based on self-assessment scales, which may be influenced by social desirability. (3) All samples are sourced from China, and the research results may be influenced by the unique social and cultural context as well as the structural background of DanceSport programs in China, which could affect the international applicability of the study, however, this has also adapted to a certain extent to mixed-gender pair sports, in which there are fewer males and more females, such as ice dance, competitive aerobics mixed doubles, artistic swimming mixed doubles and so on.

## 7 Future research directions

This study confirms that passion is the core driving force behind the DSE of university students specializing in DanceSport. Although differences in dopamine levels may partially explain gender differences, social role theory emphasizes that men’s technical advantages in DanceSport may stem from gender inequality in the allocation of early training resources (such as men being more likely to receive attention from coaches). Future research could employ longitudinal designs to separate biological and socialization effects. Additionally, the “positive” prediction of passion for women’s DSE may obscure implicit costs (such as the risk of burnout); future studies could use experimental designs to examine the intervention effects of partner support on the passion-DSE pathway for women. Finally, the impact of cultural differences between the East and West on gender moderation effects could also be explored.

## Data Availability

The original contributions presented in the study are included in the article/supplementary material, further inquiries can be directed to the corresponding author.
